# A thematic analysis of factors influencing recruitment to maternal and perinatal trials

**DOI:** 10.1186/1471-2393-8-36

**Published:** 2008-08-07

**Authors:** Rebecca L Tooher, Philippa F Middleton, Caroline A Crowther

**Affiliations:** 1 Discipline of Obstetrics and Gynaecology, University of Adelaide, Adelaide, Australia

## Abstract

**Background:**

Recruitment of eligible participants remains one of the biggest challenges to successful completion of randomised controlled trials (RCTs). Only one third of trials recruit on time, often requiring a lengthy extension to the recruitment period. We identified factors influencing recruitment success and potentially effective recruitment strategies.

**Methods:**

We searched MEDLINE and EMBASE from 1966 to December Week 2, 2006, the Cochrane Library Methodology Register in December 2006, and hand searched reference lists for studies of any design which focused on recruitment to maternal/perinatal trials, or if no studies of maternal or perinatal research could be identified, other areas of healthcare. Studies of nurses' and midwives' attitudes to research were included as none specifically about trials were located. We synthesised the data narratively, using a basic thematic analysis, with themes derived from the literature and after discussion between the authors.

**Results:**

Around half of the included papers (29/53) were specific to maternal and perinatal healthcare. Only one study was identified which focused on factors for maternal and perinatal clinicians and only seven studies considered recruitment strategies specific to perinatal research. Themes included: participant assessment of risk; recruitment process; participant understanding of research; patient characteristics; clinician attitudes to research and trials; protocol issues; and institutional or organisational issues. While no reliable evidence base for strategies to enhance recruitment was identified in any of the review studies, four maternal/perinatal primary studies suggest that specialised recruitment staff, mass mailings, physician referrals and strategies targeting minority women may increase recruitment. However these findings may only be applicable to the particular trials and settings studied.

**Conclusion:**

Although factors reported by both participants and clinicians which influence recruitment were quite consistent across the included studies, studies comparing different recruitment strategies were largely missing. Trials of different recruitment strategies could be embedded in large multicentre RCTs, with strategies tailored to the factors specific to the trial and institution.

## Background

Difficulty with recruitment to randomised controlled trials is a significant obstacle to their successful completion. Trials frequently fail to recruit the number of participants required or require extensions of the recruitment period. A recent study suggests as few as one third of UK trials recruited the required sample size in the planned period for recruitment and another third needed to extend the recruitment period [1]. Such trials may then be underpowered to detect clinically meaningful differences in important outcomes [2], substantially reducing trial precision [3]. If the recruitment period is extended in order to reach the target it is possible that clinical practice may change before the results of the trial become available [2,4]. Problems with recruitment can also lead to selective enrolment, reducing the generalisability of trial results [3].

Randomised trials in perinatal medicine face some additional hurdles to successful recruitment. Clinical outcomes of importance may be rare, therefore very large sample sizes are required to detect significant differences in health outcomes for the mother or baby [5]. Consequently, many maternal and perinatal trials need to be multicentre, adding additional complexity to the recruitment task. The need for large sample sizes may also result in situations where the same women and their babies are asked to participate in more than one trial. However, consent for maternal and perinatal trials is often challenging as women and parents are very vulnerable at the time consent is required and may have difficulty in making fully informed decisions [5,6].

We reviewed the literature regarding recruitment to maternal and perinatal trials in order to identify barriers and enablers to successful recruitment and strategies which may be effective in enhancing the recruitment effort. This literature review was used to provide an evidence resource for two workshops based on recruitment convened by the WOMBAT (Women and Babies Health and Wellbeing: Action Through Trials) Collaboration in November 2006 and March 2007.

## Methods

### Literature review

We searched MEDLINE and EMBASE from 1966 to December Week 2 2006 and hand searched reference lists of relevant articles and conference proceedings for studies of any design, including qualitative research, which focused on recruitment to perinatal trials. We also searched the Cochrane Library Methodology Register in December 2006. Studies were included in the review if they obtained data from either participants (women and/or parents), clinicians, or others involved in the recruitment of participants for perinatal trials. Studies which focused on the consent process were considered for inclusion, as recruitment and consent in maternal and perinatal research may be closely linked. If no studies of maternal or perinatal research could be identified, studies which focused on recruitment to trials in other areas of healthcare were also included, if it was felt the information would be relevant to maternal and perinatal trialists. Studies of nurses' and midwives' attitudes to research in general were included as no studies specifically about trials were located. Papers which reported primarily anecdotal evidence, opinion or commentary were excluded, as were papers which did not directly add to the body of evidence collated. Such exclusions were determined by consensus after discussion between the authors. Only papers in English were included. One author reviewed all titles and abstracts and identified potentially eligible papers for inclusion in the analysis. These were then checked by a second author for relevance and any disagreements were resolved by discussion. Search terms included subject* or patient* recruit*, enrol*, participat*, enlist*, trial*, study, research, pregnancy, childbirth, neonat*, obstetric*.

Demographic details and results for each study were extracted by one author, and checked by a second. As there were no data suitable for statistical pooling, we synthesised the data in narrative form, using a thematic analysis [7], with themes derived from the literature and after discussion between the authors. A thematic analysis "...involves the identification of prominent or recurrent themes in the literature, and summarising the findings of the different studies under thematic headings" [7] (p.47).

### WOMBAT Workshops on recruitment to maternal and perinatal trials

Half-day workshops were held in November 2006 in Sydney and March 2007 in Melbourne. Participants were trialists with a range of experience from novice to expert. This literature review was used as reference material by presenters who focused on Hot Topic areas in recruitment to perinatal trials. These presentations provided a foundation for the subsequent small group discussion on factors that influence successful recruitment and strategies to improve recruitment. Outcomes of the small group discussion were reported back to all the workshop participants and collected by the authors as a resource for future trialists. These results are reported below.

## Results

### Results of the literature search

The studies included in the review have been summarised in Table 1. We included 53 studies, of which 22 used a questionnaire design, 11 used a qualitative design, 11 were systematic or other reviews and 9 reported recruitment data. There were 21 studies which focused on participant (women/families or babies) factors (see Table 2) and 24 studies which focused on clinician factors (see Table 3). We identified eleven studies which considered strategies to improve recruitment, including four systematic reviews. Although more than half of the included studies (29/53) were specific to the maternal and perinatal healthcare context, we identified only one study which focused on barriers and enablers for clinicians working in maternal and perinatal medicine [8] and only seven studies considered recruitment strategies specific to maternal and perinatal research: two reviews [9,10]; one cluster RCT [11]; three observational studies [12-14]; and one before and after design.[15]

**Table 1:** Summary of papers included in the literature review*

**Study topic**	**Systematic and other reviews**	**Questionnaires/surveys**	**Qualitative studies**	**Recruitment data recorded**
Participants factors (Women)		**East 2006 **[30] n = 600 women	**Baker 2005 **[22] n = 17 women	**Jefferies 2006 **[31] n = 48 women
		**Rodger 2003^a ^**[21] n = 50 women	**Daniels 2006 **[29] n = 262 women	
		**Vnuk 2000 **[32] n = 106 women	**Kenyon 2006 **[16] n = 22	
			**Mohanna 1999 **[24] n = 18 women	
			**Rodger 2003^a ^**[21]	

Participants factors (Babies)		**Burgess 2003 **[17] n = 72 parents	**Hoehn 2005 **[34] n = 34 parents	
		**Morley 2005 **[35] n = 50 mothers and 48 fathers	**Mason 2000 **[18] n = parents of 200 infants	
		**Singhal 2002 **[23] n = 52 parents of babies in NICU and 106 parents of babies in normal nursery	**Snowdon 1999 **[33] n = 44 parents	
		**Zupancic 1997 **[20] n = 140 parents	**Snowdon 2006 **[19] n = 78 parents	

Clinician factors (Doctors)	Rendell 2006 [48] Cochrane review of 11 observational studies	**Singhal 2004^b ^**[8] n = 64 doctors		
		Caldwell 2005 [26] n = 250 paediatricians and 250 physicians		
		Fallowfield 1997 [25] n = 357 oncologists and surgeons		
		Somkin 2005 [27] n = 199 oncologists		

Clinician factors (Nurses/midwives)		**Singhal 2004^b ^**[8] n = 50 nurses	**Meah 1996 **[44] n = 32 midwives	
		**Hicks 1995 **[41]** Hicks 1995 **[42] n = 395 midwives	Brown 2002 [28] n = 27 women recruiters	
		**McSherry 1997 **[45] n = 297 nurses and midwives	Roxburgh 2006 [46] n = 7 nurses	
		Adamsen 2003 [40] n = 79 nurses		
		Kuuppelomaki 2003 [43] n = 400 community nurses		
		Watson 2005 [47] n = 485 staff		

Strategies to improve recruitment	**Gates 2004 **[9] n = 0 studies located	**Duggal-Beri 2006 **[11] n = 27 nurses and 22 medical staff		**Homer 2000 **[12] n = 1089 women
	**Hundley 2004 **[10] n = 11 studies			**Kenyon 2000 **[15] n = 64 UK maternity hospitals (total births 230000–240000
	Bryant 2005 [57] n = 3 cross-sectional surveys			**Moore 1997 **[13] n = 3159 women eligible for study
	Mapstone 2002 [58] n = 15 RCTs or quasi-RCTs			**Peindl 2003 **[14] n = 283 and 306 women screened
	Watson 2006 [59] n = 14 studies			

Trials in other areas of healthcare	Abraham 2006 [39] n = 94 studies of recruitment to surgical trials	Abraham 2006 [49] n = 18 surgeons and 113 patients		Gillan 2000 [4] n = 2 trials
	Fayter 2006 [52] n = 56 studies of cancer trials	Ling 2000 [50] n = 1206 patients suitable for palliative care trials		McDonald 2006 [56] n = 114 trials
	McDaid 2006 [55] n = 8 studies of cancer trials	Sullivan 2004 [51] n = 16 health professionals		

Minority populations	Bartlett 2005 [36] n = 52 trials and 134598 patients (record linkage)	Hussain-Gambles 2004 [53] n = 20 health professionals and 75 patients		**Ruggiero 2003 **[37] n = 958 eligible women
	Wendler 2006 [54] n = 20 studies of consent decisions			Rochon 2004 [38] n = 280 RCTs

*Perinatal specific papers are shown in bold type. a – Rodger 2003 reports both questionnaire and interview data; b – Singhal 2004 reports data for doctors and nurses.

**Table 2:** Participant factors which influence recruitment

**Themes and specific findings**
**Understanding of risk**
▪ Women and parents may underestimate the risks involved in trial participation and overestimate the benefits [16-19,23]
▪ Typically risks to the baby dominate over risks to the mother in decisions to participate in trials [19,21-24]

**Recruitment process and procedures**
▪ The process of recruiting women and babies into trials has an impact on the decision to participate [17,18,22,23]
▪ Communication skills of recruiters are important both to those recruiting and to potential research participants [16,22,24-29]
▪ There is conflicting evidence about the provision of written information such as patient information sheets [16-18,22,24]
▪ The timing and method of approach may impact on women's and parent's decision to participate [17,19,22,30]
▪ Practical issues faced by potential participants include:
- work and childcare commitments[28,36]
- transport issues [28,36]
- privacy and confidentiality concerns [28]
- practical concerns about the trial medications/treatments [22,28,31]

**Participants understanding of the research process and methodological issues**
▪ Women and parents may not understand specific elements of trial design such as randomisation, blinding and the use of placebos [8,16,17,21-23,32,33]

**Patient characteristics**
▪ Characteristics of patients may be related to women's and parent's decisions to participate in research:
- altruism [20,22,23,28,34,35]
- attitudes and beliefs about research [28,36]
- cultural background [12,37,38]
- language barriers [12,37,38]

**Table 3:** Clinicians factors which influence recruitment

**Themes and specific findings**
**Clinicians' attitudes to research and trials**
▪ Clinicians who are more research oriented are more likely to be involved in trials and to recruit participants [8,26,39]
▪ Although nurses and midwives typically report moderate to strong research orientation this does not generally translate into research activity or involvement mainly due to lack of sufficient research training and insufficient time for research [41-47]
▪ Doctors who believe trial participation affects patient-doctor relationship are less likely to recruit patients into trials [48]
▪ Doctors choosing not to participate in trials may believe that trial participation restricts ability to individualise care [26]
▪ Doctors with a strong preference for one or other therapy are less likely to enrol patients in trials [39,49]
▪ Evidence unclear whether discussing uncertainty is a barrier to recruitment for clinicians [9,26,39,48]

**Issues related to the trial protocol and methodology**
▪ Aspects of trial protocol can affect clinicians' participation in trials and recruitment activity [26,27,39,50,51]
- treatments more aggressive than standard
- use of placebo
- complex protocols
- strict eligibility criteria
▪ Relevance of trial, especially local relevance [27,39,52]

**Clinician beliefs about potential participants**
▪ Younger patients with better prognosis are more likely to be asked to enrol [39,48]
▪ Women and babies from minority groups are less likely to be recruited [53,54]
▪ Women and babies thought to be unable to participate or to consent are less likely to be recruited [10,39,50,52]

**Organisational/institutional issues**
▪ Lack of time is a significant barrier to trial involvement for doctors and nurses [9,25-27,39,41-43,45,46,52,54]
▪ A positive organisational culture and material support for trial activity increases clinician participation [9,26,27,39,41-47]
▪ A range of practical barriers to setting up and running a trial have been identified [9,10,14,39,50,52,55,56]
- identifying and contacting eligible patients
- ethics approvals
- setting up trial treatment procedures

### Quality of included studies

As the majority of papers included in this literature review were descriptive studies of factors which influence recruitment we have not formally assessed their quality, as there is no generally accepted or validated method of doing so for these study designs. Eleven studies focused on strategies to improve recruitment and hence tested an intervention of some type. The four systematic reviews were of good quality but were limited in their conclusions by the poor quality of the studies included in them and the poorly populated evidence base (so that many of strategies considered were only tested in one or two RCTs). The seven studies which focused on recruitment strategies for maternal and perinatal trials were limited by the designs of the studies used. Although Duggal-Beri [11] was a cluster RCT, the results reported were in abstract form only and did not provide data regarding changes in recruitment rates. One of the two reviews [9], was a commentary and therefore did not provide details of search strategies or inclusion criteria; and the other [10] provided insufficient methodological detail to confidently rule out bias. The other four studies [12-15] did not use a control group for comparison and therefore it is difficult to determine the relative effectiveness of the strategies employed.

### Participant factors which influence recruitment

#### Perception of risk

Participant assessment of risk is very important in terms of the decision to consent or refuse participation in RCTs; both for women in deciding about their own participation, and for parents in deciding about the participation of their baby. Participants (particularly parents) frequently underestimate the risks their baby might face by participating in a perinatal randomised controlled trial. A number of studies found that parents thought there would be minimal or no risk for their baby in trial involvement [16-19]. Giving consent and the speed of decision making have also been found to be related to perceptions of low or no risk for the baby [19,20]. In making the decision to participate women and parents weigh up the risks of participation against the possible benefits. Typically, the mother's/parent's duty to their (sometimes unborn) child will be given a higher priority than either consideration of the mother's own health [21] or any altruistic motives that the woman or family may have about research participation [22,23]. When risk is perceived to be too high consent will typically be refused [21,24] possibly with a resultant loss of trust in the doctors providing care [19].

#### Recruitment process and procedures

The processes by which recruitment is achieved can have an important bearing on women's decisions to participate in maternal and perinatal trials. Recruiters, in particular those also providing women's clinical care, should be aware that women and parents may feel vulnerable and coerced by the recruitment process [17,22,23]. Parents may feel pressured to make a decision quickly, and fear the baby will receive less than optimal care if consent is refused [18,22]. The communication skills of recruiters have been found to impact on the success of recruitment in terms of providing information about a trial to potential participants, obtaining informed consent and discussing uncertainty [23-27]. Developing a personal relationship with the study participant and individualising the recruitment approach for each woman or family may facilitate recruitment and ongoing involvement in research [19,22,28,29].

The timing and method of approach also has important implications for the success of recruitment. Consideration of women's situations before presenting research information should ensure that requests for trial participation are not made when a woman is in a particularly vulnerable position [22]. Women and parents may prefer to have information earlier, or have more time to consider their decision to participate in the trial [17,30]. The provision of written information such as Patient Information leaflets may assist in recruiting participants [18,22,24], ideally used together with verbal information to support the relationship between the recruiter and participant [17,22,24]. Careful consideration given to the content of information sheets, with excessive jargon avoided where possible, may also enhance recruitment [17].

A range of practical issues which impact on participation were also identified. These included work and childcare commitments, holiday plans, and transportation issues [22,28,31]. Privacy and confidentiality concerns in small communities may also be a barrier to involvement in research [28]. Other practical barriers relate to trial treatment schedules and medications [21,32].

#### Participants' understanding of the research process and methodological issues

While potential participants may understand the purpose of the research and the procedures involved, many do not appear to understand why a randomised design has been used or what the implications of this may be [8,17,22,23,33]. Specific elements such as randomisation, blinding, and the use of placebos may be poorly understood, with participants demonstrating a preference for designs which are unblinded [16], do not include a placebo [21], or do not involve random allocation to treatment [32].

#### Individual beliefs and attributes related to decision to participate

A range of personal beliefs and attributes may be related to the decision of women and parents to participate in randomised controlled maternal and perinatal trials. Altruism is commonly reported as a reason for research participation [20,22,23,28,34,35]. Positive or negative beliefs about research including the level of trust held in research and clinical governance also influence trial participation [28,36]. Cultural background and language barriers have also been found to influence the participation of women from minority groups [12,37,38].

### Clinician factors which influence recruitment

#### Clinicians' attitudes to research and trials

There is a relationship between a clinician's research orientation and their research involvement including recruitment to clinical trials [25,26]. Higher research orientation is generally correlated with research experience, research involvement and recruitment to trials [8,26,39]. Although nurses and midwives typically report a moderate to strong research orientation this does not always translate into research activity or involvement mainly due to lack of sufficient research training and insufficient time for research activities [40-47].

Doctors' attitudes and beliefs about trials may affect trial participation and recruitment. Doctors who believe trial participation affects the patient-doctor relationship are less likely to recruit [48]. Doctors who believe trial participation restricts their ability to individualise patient care are less likely to participate in trials [26], as are doctors with a strong preference for one of the treatment arms [39,49]. Clinicians who are motivated to participate in a clinical trial by a personal relationship with the investigator(s) are less likely to recruit than those motivated by other factors [48], suggesting some clinicians may feel pressured to agree to trial participation by their personal acquaintance with researchers, without a having a strong commitment to the trial question or processes involved. Doctors' handling of uncertainty may affect trial participation and recruitment, however, the evidence is conflicting [9,26,39,48].

#### Issues related to the trial protocol

Aspects of the trial protocol can affect clinicians' participation and recruitment activity. Trials involving treatment more aggressive than the standard treatment, trials involving a placebo, complex trial protocols (that require extra time to learn about eligibility and treatment), and strict eligibility criteria have all been cited as barriers to trial participation by clinicians [26,27,39,50,51]. Relevance of the trial, especially local relevance, was also identified as barrier to participation by clinicians [27,39,52]. Trials with more pragmatic designs in line with standard practice and that were easier to explain to patients and logical extensions of previous trials may increase clinicians' involvement [52].

#### Clinician beliefs about potential trial participants

Patient characteristics may affect clinicians' decisions to offer trial participation or recruit eligible patients. Younger patients and those with better prognosis are more likely to be invited to participate [39,48]. Patients who clinicians believe have higher intelligence are also thought to be easier to communicate with about trials [25]. Clinician gate-keeping of patients judged to be unable to participate in a trial, or provide informed consent at the time of recruitment, has also been identified as a significant barrier to recruitment [10,39,50,52]. Assumptions about the willingness of women from minority backgrounds to participate in trials has also led to an under-representation of these women in research generally and in clinical trials [53,54].

#### Institutional/organisational issues

Lack of time is a significant barrier to trial involvement for doctors and nurses/midwives. Specifically clinicians report a lack of time available for recruitment, for data management, to learn about protocol eligibility and treatment requirements, and to obtain informed consent [9,25-27,39-43,45,47,52,55]. Organisational culture and support for trials may also influence participation of clinicians and to an extent participation of women and babies in randomised trials. Lack of expert support staff to handle recruitment and data management has been cited in several studies of barriers to involvement of clinicians in randomised trials [9,27,39]. Furthermore, in one study of recruitment to 114 UK trials [56], better recruitment was significantly associated with the presence of a dedicated clinical trial manager (OR3.8, 95%CI:0.79 to 36.14, p = 0.087). Lack of financial reward, either for individuals or departments involved in trials, together with the expense and financial implications involved in trial participation has also been identified as a barrier to trial involvement [4,9,26,27]. The presence of a culture which encourages and supports randomised trial activity also impacts positively on clinicians' participation in trials. Nurses and midwives commonly report lack of support for research activities from management as a significant barrier to research involvement [41-47].

Practical barriers in setting up and running a trial can also impact negatively on recruitment. Such barriers include: identifying eligible patients; trials competing for the same patients; the need to engage and maintain the interest of the whole clinical team in the trial; and a lack of awareness of ongoing trials and eligibility criteria [52,55]. Accurate estimates of the number of patients eligible for participation in trials may also be a barrier to recruitment [56]. In trials which target or include women from minority backgrounds, investigators should keep in mind that it may be difficult to contact the women of interest [13]. Administrative barriers such as problems with staff, the ethics approval process, and implementation of study treatment procedures have also been identified as barriers to clinician involvement in trials and have been found to impact negatively on recruitment [9,56].

### Strategies to enhance recruitment to randomised trials

#### Evidence from systematic reviews

The evidence-base for strategies about improving recruitment to trials is not well populated. Although four systematic reviews were identified [55,57-59], which included 33 unique studies, few strong conclusions were able to be made. None of the reviews could identify any strategies that were clearly found to increase recruitment to trials, but conversely none of the strategies identified could be unequivocally said to be ineffective at increasing recruitment due to small numbers of studies testing each strategy and methodological weaknesses. Only one of the studies included in the four reviews addressed a maternal or perinatal topic.

A number of strategies were identified as being possibly effective in the four reviews (Table 4). However, the evidence for these strategies was either weak, or conflicting. Furthermore, conclusions about the strategies sometimes differed between the reviews due to differences in their inclusion criteria. Two of the reviews [55,57] did not identify any effective strategies. One review [59] found that recruitment was increased in trials without a placebo arm or which were not blinded. However, the other review [58] found no difference in recruitment in two studies which used no placebo or a partial randomisation design. Sending a questionnaire related to the study with the request to participate increased recruitment to a home safety trial [58,59]. A telephone reminder to non-respondents increased recruitment in one study [59]. Financial incentives were found to increase recruitment of teenage girls to a quit smoking intervention and helped to retain them in the study [58,59]. However, financial incentives to general practitioners did not increase recruitment to two primary care trials and when surveyed, financial incentives were considered of minor importance by recruiters [57]. Socioculturally specific interventions such as training lay recruiters to recruit women from particular ethnic minorities and multifaceted interventions increased recruitment in two trials, however, the increase in recruitment was small compared to the effort required [59].

**Table 4:** Summary of systematic review findings about recruitment strategies

**Strategies that might improve recruitment (but evidence weak or conflicting)**
▪ Using a trial design without blinding or a placebo group
▪ Sending a questionnaire related to the trial with the request to participate
▪ Telephone reminder to non respondents
▪ Financial incentives for participants
▪ Interventions tailored to meet the needs of particular minority groups
**Strategies not shown to significantly improve recruitment**
▪ Warning potential participants about an impending request for participation
▪ Using a personalised letter together with a flyer
▪ Changing information available to potential recruits
▪ The professional background of the recruiter (doctor vs nurse)
▪ Visiting trial sites to encourage recruitment
▪ Changes to consent process
▪ Collecting patient trial data by internet vs paper methods

This table summarises results from four systematic reviews which altogether considered 33 unique studies comparing different recruitment strategies. [54,57-59] The reviews all noted that there is insufficient evidence to make strong conclusions about the relative effectiveness of different strategies.

All four systematic reviews also identified a number of strategies which did not significantly alter recruitment rates. All authors highlighted the need to reserve judgement about the effectiveness of these strategies due to methodological weaknesses in the studies and the small size of the evidence base for each (often only one study).

These strategies included:

• warning potential participants about an impending request for participation

• using a personalised letter together with a flyer

• changing information available to potential recruits

• the professional background of the recruiter (doctor vs nurse)

• visiting trial sites to encourage recruitment

• changes to consent process

• collecting patient trial data by internet vs paper methods

#### Evidence about strategies to improve recruitment to perinatal trials

A cluster randomised trial using DVD training about consent for a trial of intravenous immunoglobulin for neonatal sepsis resulted in high levels of confidence and knowledge about the trial [11]. The study was reported only as an abstract and did not report effects on recruitment or results of the control groups. A training intervention for midwives recruiting to a trial about antibiotics for premature rupture of the membranes (in which local midwives were employed for 3 hours per week to provide training and motivation to local staff about the trial) resulted in a significant increase in the number of women recruited out of all births (from 0.31% prior to the intervention to 0.68% after, p < 0.0001) [15]. A study of recruitment to two postpartum mental health trials (a treatment and a prevention trial) found that referral from a health professional accounted for almost half of participants for both prevention and treatment trials, and in the treatment trial 32% of participants came from mass mailing. However, media appearances and advertisements on local TV and radio resulted in many women being screened but did not translate into a large number of extra participants [14].

One non-systematic review of factors affecting recruitment to multicentre trials in maternal and perinatal health found no high quality evidence on which to base recommendations about strategies to improve recruitment [9]. It was suggested that recruitment would be improved if clinicians were trained to regard recruitment to trials as part of their normal clinical duties, and noted that the strategies used need to be targeted to the particular barriers associated with each trial or trial site. A review of recruitment to intrapartum studies found problems with all three of the most common strategies used for recruitment [10]. Antenatal recruitment involved a significant delay between enrolment and the intervention and resulted in many women being consented who would subsequently be found ineligible for trial participation. Recruitment and randomisation during labour was subject to a significant degree of clinician gate-keeping so that many potentially eligible participants were not approached for trial participation. Staged randomisation in which consent was gained antenatally but randomisation was done after labour commenced or at the time of the intervention resulted in substantial pre-randomisation losses. It was not possible to determine whether this was a result of participants changing their mind or clinician gate-keeping [10].

Two observational studies focused on strategies to increase recruitment of minority women (either from non-English speaking or low-income backgrounds) to maternal and perinatal trials [12,13]. Both studies found that a range of specific strategies to increase recruitment of these women resulted in high levels of participation and a sample representative of the population from which the women were drawn. Strategies included: engagement of the community; use of interpreters and translation of written materials; financial incentives; and multiple approaches and pre-warning of impending requests for participation [12,13].

#### Strategies identified at WOMBAT workshops on trial recruitment

The factors which influence recruitment and strategies for improvement that were identified in the literature are broadly similar to those collected by WOMBAT during the two workshops. Table 5 summarises strategies discussed to improve recruitment at these workshops.

**Table 5:** Summary of strategies to improve recruitment discussed in WOMBAT Collaboration workshops

**For participants:**
• provide information that is important to participants (not researchers)
• use a personalised approach in terms of method and timing of approach and request for consent to participate
• make it easy to participate by making trial protocol not too onerous
• use different methods for reaching participants including pamphlets, telephone and mass media
• increase transparency of information provided about treatment and research to help potential participants understand need for trial
• assure potential participants there will be no compromise in care if they choose not to participate
**For clinicians and participating clinical units/centres:**
• education for staff including communication skills training for potential recruiters
• provide feedback and information to all involved
• recognition of contribution through acknowledgement in any publications or where appropriate joint authorship
• incentives (usually tangible) for reaching recruitment targets
• use of mass media
• communication and support
• make recruitment easy for recruiters
• develop an important and clinically relevant question
• have multidisciplinary input (especially unit directors) into trial design to help ensure 'buy-in' from all relevant stakeholders
• reduce impact of participation on units by having a dedicated research team and a centrally funded researcher who specific role is trial recruitment
• build relationships within participating centres by identifying and nurturing local contacts and local facilitators and also people likely to influence others

**Organisational culture**
• research should be seen as 'standard care' therefore recruitment to clinical trials seen as normal part of clinical practice
• institution is committed to high quality research

This table summarises results from two workshops held by the WOMBAT Collaboration which focused on recruitment in November 2006 and March 2007.

## Discussion

There is considerable consistency in the types of factors reported by women and families which influence their participation in maternal and perinatal trials. A key theme identified was participant estimation of risk, which was found to be frequently underestimated leading them to believe that there would be little or no risk involved in trial participation. Women and parents were also found to have difficulty in understanding some of the methodological aspects of trial design, in particular the use of randomisation and placebos. It appears that parents may not clearly differentiate research and treatment [19]. This lack of understanding points to the need for better communication about trial aims and design. However, it is not possible to say whether improvements in communication of information about trials will lead to increased recruitment. Indeed, there was a suggestion from the evidence that increasing the information available to potential participants could result in fewer agreeing to participate in perinatal trials. However, better informed participants may be more likely to remain in the trial and adhere to trial treatment schedules, although this is also currently unknown.

Practical barriers to trial involvement such as difficulties with childcare and transportation appear to be relatively easily ameliorated. However, whether simple financial incentives would be sufficient to compensate women and families for trial participation remains unclear. The beliefs of minority women and families about participation in maternal and perinatal trials also require further study. Women and babies from ethnic minorities are likely to be under-represented in perinatal research including trials, despite the fact that these populations often bear a higher burden of disease for many conditions than the general population that is usually recruited for clinical trials.

The studies reviewed also suggested considerable consistency in the factors reported by clinicians which influence their recruitment efforts. Unfortunately a lack of research in this area limits our ability to determine whether there are specific issues for health professionals caring for women and their babies during and after pregnancy. It does seem that more generic issues relating to the design of the trial and some of the organisational issues identified are likely to be shared by clinicians in every area of healthcare. Some of these issues, in particular, those related to the design of the trial protocol could be addressed by investigators with relatively little additional effort, and probably no real increase in costs. In designing trial protocols, investigators could aim to use simpler rather than more complex designs and ideally develop research questions which are relevant in the localities in which the trial is to be conducted [27,39,52]. When possible, the trial design could use standard care as the basic treatment model, and limit the amount of clinical practice behaviour change which the trial would require [27,39]. Taking into consideration these types of issues at the design phase of the study may also have the added benefit of making the trial easier to manage with less risk of protocol violations.

A recruitment plan could be developed which takes into consideration the types of information to be conveyed to potential participants and the timing of requests for participation and for obtaining consent, as this has been shown to impact on women's and families' decisions to participate [17,19,22,30]. Although, we currently have no direct evidence about the impact of such a recruitment plan on the participation of clinicians in trials, it is likely that a recruitment process which fits into the standard clinical practices of the units responsible for doing the recruiting will result in more requests for participation. Investigators may need to spend some time exploring with recruiters exactly what the recruitment plan should be. A more carefully developed and streamlined recruitment process may also reduce the time demands associated with trial participation and therefore increase the willingness of clinicians to participate in trials.

Support from a clinical trial coordinator or research nurse with responsibility for trial recruitment was found to be positively linked to recruitment in two studies (one in a perinatal context) [15,56]. However, this strategy has not been tested in a randomised trial design. A supportive organisational and professional culture for research and trial activity, was suggested by many authors as a key to improving recruitment. What constitutes a positive research culture and how such a culture can be encouraged remains undefined. Furthermore, changing organisational and professional culture is likely to be beyond the influence of most individual investigators. Here networks of researchers and clinicians are needed, with support from organisational management. Time for research is a barrier which can really only seriously be tackled by changes in the way healthcare is organised and delivered.

The extent to which maternal and perinatal trials are reliant on funding from public rather than industry or commercial sources, is likely to limit the amount of money which can be directed towards improving the recruitment effort. Therefore, it is important that research is undertaken to determine the best strategies or mix of strategies to use to improve recruitment. Trials or studies of different recruitment strategies could be nested within larger clinical intervention randomised trials. Strategies which could be tested include:

- timing of requests for consent (timing would depend on the nature of the trial);

- use of financial or other compensatory incentives for either participants and/or clinicians or hospital units;

- provision of information for participants especially about risk;

- increasing support available for trial recruitment through the provision of a recruitment officer, or protected time for recruitment.

The strategies to be tested should ideally be tailored to the factors which influence recruitment identified for a specific trial and location. A tool which assists investigators to identify these factors for both for participants and clinicians or units, would assist in the selection of appropriate strategies to include in the trial recruitment plan. A similar tool has been developed by the National Institute of Clinical Studies (NICS) in Australia for diagnostic assessment of barriers to the implementation of best available evidence into clinical practice [60].

The NICS Barrier tool enables users to work systematically through the process of identifying which people or groups of people are responsible for a particular practice and then determining what the barriers to change for each group may be. Users may work individually, or preferably in a small group, to brainstorm these issues and then identify strategies which would address the barriers identified. The Barrier Tool is accompanied by a number of information sheets and a booklet describing methods of obtaining information about barriers, including survey, consensus processes and interview techniques. The WOMBAT Collaboration is currently working on modifying the NICS Barrier Tool to create a trial recruitment tool.

## Conclusion

The factors we identified which influence recruitment for both participants and clinicians were quite consistent across the included studies. However, studies which compared different strategies were largely missing from the literature. Trials of different recruitment strategies could be embedded in large multicentre RCTs, to enable assessment of this area with minimal additional effort. Ideally the strategies used should be tailored to the factors specific to the trial and institution. Such methodological research is urgently needed to provide the evidence base for effective strategies to optimise recruitment into randomised trials.

## Competing interests

The authors declare that they have no competing interests.

## Authors' contributions

RT participated in the design of the study, carried out data collection and data extraction, performed the data synthesis, and drafted the manuscript. PM carried out data extraction and both PM and CC participated in the design of the study, the interpretation of data and critically revised the manuscript for important intellectual content. All authors have given final approval of the version to be published.

## Pre-publication history

The pre-publication history for this paper can be accessed here:


